# Effect of NaCl Reduction on Dough Rheology and Bread Quality of Fibre-Enriched White Wheat Bread

**DOI:** 10.3390/foods15132343

**Published:** 2026-07-02

**Authors:** Sabrina Boudrag, Elke K. Arendt, Emanuele Zannini

**Affiliations:** 1 School of Food and Nutritional Sciences, University College Cork, College Road, T12 YN60 Cork, Ireland; 124100939@umail.ucc.ie (S.B.); e.zannini@ucc.ie (E.Z.); 2 APC Microbiome Ireland, Biosciences Building, University College Cork, T12 YT20 Cork, Ireland; 3 Department of Environmental Biology, Sapienza University of Rome, Piazzale Aldo Moro 5, 00185 Rome, Italy

**Keywords:** sodium chloride, dietary fibre, salt/sodium reduction, wheat bread, wholemeal

## Abstract

Excessive dietary salt intake remains a leading preventable risk factor for cardiovascular disease and mortality. Bread is recognised as a major contributor to population-level sodium consumption in Europe and globally. This study investigated whether substantial salt reduction in bread can be achieved without compromising dough rheology, gas retention, or final bread quality across diverse flour matrices. We evaluated four sodium chloride levels (1.2, 0.6, 0.3, and 0% *w*/*w*) in three wheat-based systems: refined white flour (control), fibre-enriched white flour, and wholemeal flour. Dough properties were characterised using GlutoPeak analysis (gluten network development) and Rheofermentometer testing (yeast fermentation kinetics). Bread quality was assessed through measurements of specific volume, crumb texture, cell structure, staling rate, and water activity. Across all flour types, 0.6% salt (*w*/*w*) emerged as a technologically optimal concentration, providing superior balance between dough functionality and bread quality while achieving a 50% reduction relative to conventional industrial formulations (1.2% *w*/*w*). This optimal salt level maintained acceptable technological performance while conferring a significant public health benefit through sodium reduction. Additionally, we successfully developed a fibre-enriched white wheat bread formulation combining 0.6% salt with enhanced dietary fibre content (9.3 g/100 g vs. 3.8 g/100 g in conventional bread), representing a synergistic dual-reformulation strategy addressing two major dietary insufficiencies simultaneously: excessive sodium and inadequate fibre intake. These findings demonstrate that substantial salt reduction in bread, a daily-consumed staple food, is both technologically feasible and nutritionally compelling, offering industry a clear pathway for population-level dietary improvement.

## 1. Introduction

Salt, the common term for sodium chloride (NaCl), is an essential dietary mineral that contributes to fluid and electrolyte balance, nerve transmission and muscle contraction in humans. It provides around 90% of the sodium in the human diet [1]. Despite its physiological importance, salt intake in many European populations exceeds recommended levels, primarily due to the widespread use of salt in processed foods [2]. Excess sodium intake is strongly associated with elevated blood pressure and an increased risk of cardiovascular diseases, and population-wide salt reduction has therefore been identified as a cost-effective strategy for improving public health [3,4]. In Europe, most health authorities and international organisations recommend limiting intake to about 5–6 g salt per day (corresponding to roughly 2 g sodium) for adults, to reduce the risk of hypertension and cardiovascular disease [2,4,5]. However, recent assessments show that mean salt intakes across the WHO (World Health Organization) European Region almost always exceed these values, with most countries reporting average intakes well above 7.5 g/day and up to 18 g/day in some populations [6]. For adults, WHO recommends <2000 mg/day of sodium (<5 g/day salt) [7]. Within this context, cereal products, and bread in particular, are recognised as major contributors to daily salt intake, making them a critical target for sodium-reduction policies [3,8]. Conventional white wheat bread typically contains around 1.2% salt (*w*/*w*) in many industrial and research formulations, a level that reflects both technological requirements and consumer expectations for flavour [1,8,9]. In 2021, the WHO developed global benchmarks for sodium levels across different food categories, in order to help countries set national policies. The recommended levels for leavened bread were set at 330 mg sodium/100 g of bread, around 0.8 g salt/100 g bread [10]. Under Regulation (EC) No 1924/2006, the European Union defines several nutrition claims related to salt/sodium [11]. A food may be labelled “low sodium/salt” if it contains no more than 0.12 g sodium per 100 g, equivalent to about 0.3 g salt per 100 g. “Very low sodium/salt” is restricted to products with no more than 0.04 g sodium per 100 g (0.1 g salt per 100 g), while “sodium-free/salt-free” may be used only when the sodium content does not exceed 0.005 g per 100 g (0.01 g salt per 100 g). In addition, a “reduced sodium/salt” claim is permitted when the sodium (or salt) content in a product is at least 25% lower than that of a comparable reference product. Recent reformulation efforts have focused on reducing salt content in line with nutritional recommendations while maintaining product quality and consumer acceptance [1,3,12,13,14]. However, reducing salt in bread is not trivial because sodium chloride fulfils multiple technological roles in dough and bread systems. It strengthens the gluten network and modifies dough viscoelasticity; it slows yeast fermentation via osmotic effects; and it influences water mobility, starch–protein interactions and, indirectly, crumb structure and staling behaviour [9,15,16,17,18]. Salt also contributes to flavour and may modulate Maillard-derived aroma and colour development during baking [19,20]. Given these functional and sensory roles, a key challenge in salt reduction is identifying sodium levels that reduce salt intake without compromising dough performance or bread quality. While numerous studies have evaluated NaCl reduction in conventional white wheat breads, much less is known about how sodium behaves in fibre-fortified doughs, where added dietary fibres and bran components alter gluten development, water distribution and the local ionic environment. There is increasing evidence that dietary fibres can interact with charged solutes (including ions and bile salts) through electrostatic and related physicochemical interactions, affecting solute binding, water structure and rheology [21,22]. On this basis, we hypothesised that (1) reducing salt from 1.2% to intermediate levels would affect dough rheology and bread quality differently in fibre-fortified and wholemeal matrices than in refined white wheat, and that (2) in fibre-enriched systems, NaCl may interact with dietary fibres via electrostatic and associated interactions, contributing to an attenuated or altered salt responsiveness compared with the white wheat control. Understanding whether an optimal salt level can be identified across such diverse formulations is therefore of both technological and nutritional interest.

In a previous study, we developed an optimised fibre-fortified white wheat bread formulation containing a combination of soluble (beta-glucan and pectin) and insoluble dietary fibres (oat fibre and cellulose), whose technological and sensory characteristics were comparable to those of conventional white wheat bread, despite its higher fibre content, which is comparable to that of wholemeal bread. The selection of this mixed-fibre blend was underpinned by the complementary physiological benefits of its components, particularly the role of insoluble fibres in promoting gut motility and that of soluble fibres in regulating postprandial glucose and serum cholesterol levels [23]. Building on this formulation, the present study aims to reduce its salt content in order to produce bread with improved nutritional quality, and systematically evaluates the effects of four salt levels—1.2% (*w*/*w*), representing a conventional level in bread; 0.6% (*w*/*w*), corresponding to a 50% salt reduction; 0.3% (*w*/*w*), classified as low-salt bread according to regulatory criteria; and 0% (*w*/*w*) serving as a salt-free control—on dough and bread properties in three wheat-based systems: white wheat (control), fibre-fortified white wheat, and wholemeal.

By combining measurements of dough behaviour (gluten aggregation and yeast fermentation) and key bread quality attributes (including specific volume, texture, crumb structure, colour and water activity), our aim is to determine how salt interacts with different flour matrices, especially with dietary fibres, and to identify salt levels that provide an acceptable compromise between technological performance and the public-health objective of sodium reduction, providing practically relevant guidance for sodium reduction in fibre-rich formulations. Although this work focuses on dough and bread technological properties, successful salt reduction ultimately depends on sensory acceptance, since it is a primary contributor to flavour and overall liking; therefore, the present study should be viewed as addressing the technological feasibility dimension, with consumer validation needed in future work.

## 2. Materials and Methods

### 2.1. Raw Material

Ingredients used for the experiments included refined white wheat baker’s flour (BF) and fine wholemeal flour (WMF) (Odlums Group, Dublin, Ireland). The dietary fibre sources used for the optimised recipes were VITACEL^®^ L 600-30 cellulose from citrus peel (JRS, Rosenberg, Germany); VITACEL^®^ Oat Fibre HF 401 (JRS, Germany); GENU^®^ Pectin type BIG (CP Kelco, Atlanta, GA, USA); and PromOat^®^ Beta-Glucan from oat grain (Lantmännen Oats AB, Stockholm, Sweden). Vital gluten (Roquette, Lestrem, France) was incorporated into the fibre-fortified white wheat recipes to counter protein loss from BF substitution. Additional baking ingredients included salt (Glacia British Salt Limited, Cheshire, UK), sugar (Siúcra, Dublin, Ireland), instant active dried baker’s yeast (Bruggeman Brown Yeast, Puratos, Groot-Bijgaarden, Belgium), sunflower oil (Musgraves, Cork, Ireland) and tap water at 25 °C. Table 1 summarises all 12 formulations.

### 2.2. Water Content Adjustment

The water content of each formulation was optimised using a Farinograph-TS^®^ (Brabender GmbH & Co. KG, Duisburg, Germany) in accordance with the AACC International Standard Method 54–21 [24]. A 300 g sample of flour ingredients and salt (at 14% moisture basis) was first mixed for one minute in the instrument’s mixing chamber to ensure uniformity prior to water addition. Water was then automatically dosed to reach a target dough consistency of 500 ± 20 Farinograph Units (FU), while maintaining the chamber temperature at 30 °C. The Dough Development Time (DDT) was also recorded. All measurements were conducted in triplicate. For each flour–salt formulation, the amount of added water was adjusted so that the dough reached the standard Farinograph reference consistency of 500 ± 20 FU, ensuring that all rheological parameters were measured at a comparable dough firmness.

### 2.3. Compositional Analysis of Flour and Bread

The compositional analysis of the flours and breads was performed by an external accredited laboratory (Chelab S.R.L., Mérieux NutriSciences Corporation, Resana TV, Italy). Moisture was assessed using a method based on AOAC 950.46 B 1991 and AOAC 952.08 1961 [25,26]. Sodium content was determined using a method based on the standard method AOAC 2011.14 [27]. Protein content was determined based on AOAC 990.03, AOAC 992.15, and AOAC 992.23 [28,29,30]. Fat was quantified by an internal method reported as MP 2696 rev 0 2025. Ash was measured in accordance with AOAC 945.46, AOAC 923.03, AOAC 938.08, and AOAC 920.93 A [31,32,33,34]. Carbohydrate content was obtained by subtracting the total of the other nutrients, using the procedure described in AOAC 986.25 [35]. The Total Dietary Fibre was measured following AOAC 2017.16 [36].

### 2.4. Characterisation of Dough Properties

#### 2.4.1. Dough Preparation

The dry ingredients, including flour, dietary fibres, salt, and sugar, were premixed. Yeast was rehydrated in the total volume of water at 25 °C for 10 min and incorporated into the dry mixture along with sunflower oil. Fibre-enriched formulations included gluten to compensate for protein loss. Mixing was performed using a Kenwood Chef Classic mixer (Kenwood Manufacturing Co., Ltd., Havant, UK) equipped with a dough hook, operating at speed 1 for 1 min. The bowl sides were then scraped down and mixing continued at speed 2 for a further 7 min.

#### 2.4.2. Gluten Network Development

Gluten network development was assessed using a Brabender GlutoPeak (Brabender GmbH & Co. KG, Duisburg, Germany). First, 9 g of flour ingredients and salt (calculated on a 14% moisture basis) was dispersed in deionised water at 36 °C to obtain a total sample weight of 18 g. Torque was continuously recorded at 36 °C under a shear rate of 2750 rpm. The Peak Maximum Time (PMT, s) and maximum torque (TM in Brabender Units, BU) were determined from the resulting torque–time curve.

#### 2.4.3. Dough Fermentation

Dough fermentation characteristics were assessed using a Rheofermentometer F3 (Chopin Technologies, Villeneuve-la-Garenne CEDEX, France). First, 300 g of dough was placed in the fermentation chamber and covered with a 1500 g cylindrical weight. The chamber was hermetically sealed, and fermentation proceeded for 3 h at 30 °C. During the test, the maximum dough height (HM, mm), the total volume of CO_2_ released (mL) as well as the volume of CO_2_ lost (mL) were continuously recorded.

#### 2.4.4. Breadmaking

Following dough preparation, 450 g of dough was transferred into a greased baking tin (15 × 9.5 × 9.7 cm) and proofed for 90 min at 35 °C and 75% relative humidity using a KOMA SunRiser proofer (KOMA, Roermond, The Netherlands). The proofed dough was then baked in a preheated deck oven (MIWE Condo, Arnstein, Germany) at top and bottom temperatures of 220 °C and 230 °C, respectively, following a 400 mL steam injection. Baking was carried out for 35 min, after which the loaves were cooled at room temperature for 2 h prior to analysis.

### 2.5. Bread Quality Evaluation

Specific volume was measured on whole, uncut loaves. For all other analyses, a single loaf was sliced into five 25 mm thick sections, excluding the end pieces. For each formulation, three independent baking trials were performed; in each trial two loaves were produced.

#### 2.5.1. Specific Volume

The specific volume of the bread loaves (mL/g) was measured using a Volscan Profiler (Stable Micro Systems, Surrey, UK).

#### 2.5.2. Crumb Structure

A C-Cell Imaging System (Calibre Control International Ltd., Warrington, UK) was used to quantify crumb cell number and diameter, following the standardised protocol supplied by the instrument manufacturer for bread imaging analysis.

#### 2.5.3. Colourimetry

The surface colour of the bread crumb was assessed using a Minolta Chroma Meter CR-410 (Konica Minolta Holdings Inc., Osaka, Japan) operating in the CIE L*a*b* colour space (Commission Internationale de l’Éclairage). Crumb colour was measured five times per slice. The colour difference (ΔE) between each sample and its corresponding salt-free control was calculated using the following equation:

(1) $$\Delta E=\sqrt{{\left({\Delta L}^{*}\right)}^{2}+{\left({\Delta a}^{*}\right)}^{2}+{\left({\Delta b}^{*}\right)}^{2}}$$

where ΔL* = L*control − L*sample, Δa* = a*control − a*sample and Δb* = b*control − b*sample.

#### 2.5.4. Textural Attributes

Texture analysis of the breadcrumb was performed using a TA-XT2i Texture Analyser (Stable Micro Systems, Surrey, UK). A double-compression test was applied to 25 mm thick bread slices using a 35 mm cylindrical probe, with 40% strain, a pre-test speed of 1 mm/s, a test speed of 5 mm/s, and a post-test speed of 10 mm/s, incorporating a 5 s interval between compressions. Breadcrumb hardness was subsequently determined from the resulting force–time curves.

#### 2.5.5. Staling Rate

The rate of staling was evaluated from the change in crumb hardness over five days of storage following the AACC 74-10.02 crumb firmness method, with minor adaptations [37]. On day 0, two loaves were prepared: one loaf was cooled for 2 h, sliced, and its crumb hardness was measured, while the second loaf was cooled and stored at room temperature in a sealed bag for five days. After storage, the second loaf was sliced, and its crumb hardness was determined. An apparent staling rate was calculated using Equation (2), providing an average rate of firmness increase between day 0 and day 5 for each formulation. This empirical staling rate is intended as a comparative index over the 0–5-day period and does not represent a detailed kinetic parameter of the staling process. A similar instrumental firmness-over-time approach has been applied previously in baked products by Sahin et al. (2018) [38].

(2) $$Staling\;rate\;\left(\%\right)=\frac{Crumb\;hardness\;at\;day\;5-Crumb\;hardness\;at\;day\;0}{Crumb\;hardness\;at\;day\;0}$$

#### 2.5.6. Water Activity

The water activity of the crumb was measured at room temperature using an Aqua Laboratory Series 3 water activity meter (Decagon devices, Pullman, WA, USA).

### 2.6. Statistical Analysis

Statistical analyses were conducted using OriginPro 2026 (OriginLab Corporation, Northampton, MA, USA). The significance threshold was set at *p* < 0.05. Data were first screened for normality using the Shapiro–Wilk test and for homogeneity of variances using Levene’s test. When both assumptions were met, one-way ANOVA was used to evaluate the effect of salt level on dough and bread characteristics, followed by Tukey’s HSD post hoc test for pairwise comparisons. When normality was violated but variances were homogeneous, the Kruskal–Wallis test was applied, followed by Dunn’s post hoc test with Bonferroni correction for multiple comparisons. When homogeneity of variances was not met, Welch’s ANOVA was used together with the Games–Howell post hoc test. For all post hoc procedures, *p*-values < 0.05 were considered statistically significant.

## 3. Results

### 3.1. Water Content Adjustment

The optimal water content of each formulation was determined to achieve the desired dough consistency. The Farinograph^®^ data are presented in Table 2. Among the tested samples, the white wheat control formulations showed the lowest water absorption, whereas the fibre-enriched white wheat samples exhibited the highest. Across all flour types, higher salt addition led to lower water absorption and higher DDT. Similar findings were made in other studies looking at the effects of salt reduction on the properties of wheat dough [16,39]. These data were then used to formulate the recipes; the reduced-salt formulations (0.6% salt; *w*/*w*) are reported in Table 3, where this intermediate salt level was selected for presentation to avoid the excessive complexity of displaying all 12 recipes. For completeness, detailed formulations for the 9 other recipes are provided in the Appendix A (Table A1).

### 3.2. Compositional Analysis of Flour and Bread

The results of the flour and bread compositional analysis are presented in Table 4. Sodium contents were converted to equivalent salt contents using the conventional factor (salt [g] = sodium [g] × 2.5) specified in EU food information legislation [40].

### 3.3. Characterisation of Dough Properties

**Figure 1:**
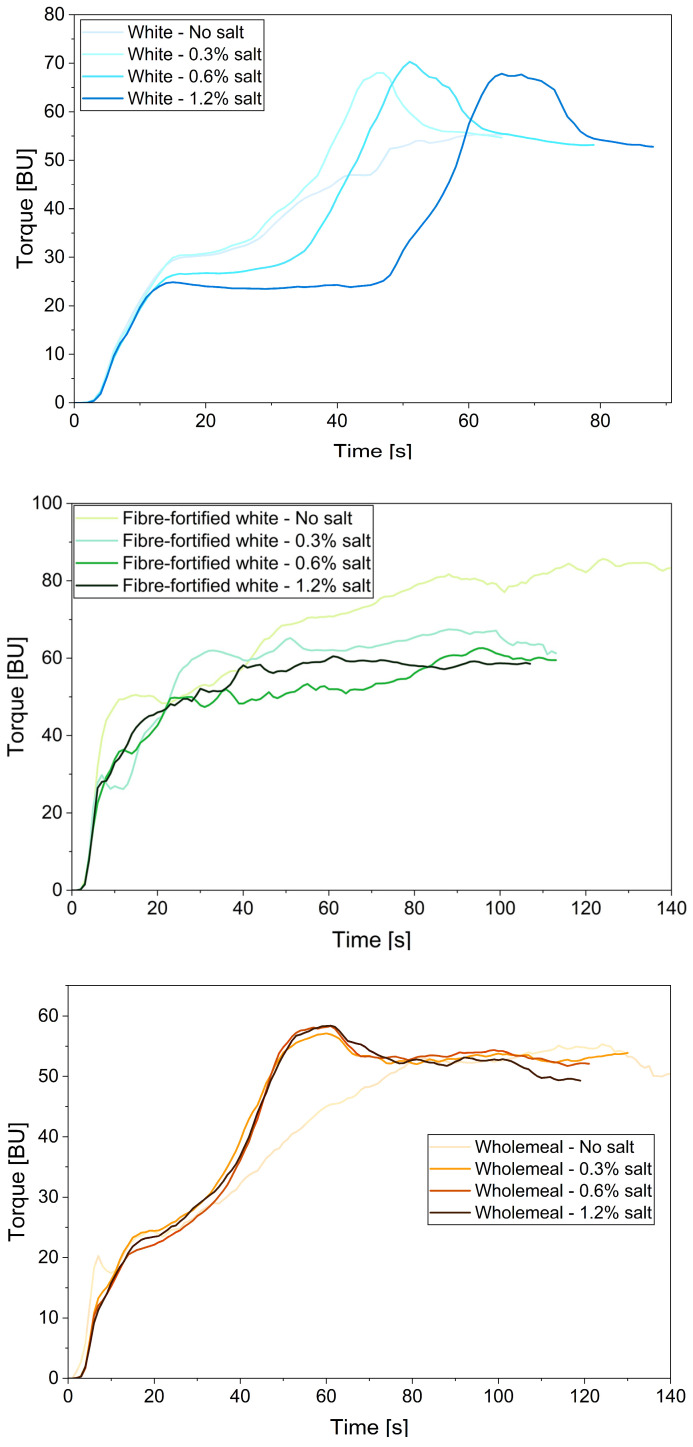
GlutoPeak curves showing gluten aggregation, expressed as torque (BU) over time (s) at 36 °C.

Gluten aggregation behaviour was characterised using the GlutoPeak test on white wheat (control), fibre-fortified white wheat and wholemeal flours containing 0 to 1.2% salt (*w*/*w*). The Peak Maximum Time PMT (s) and the Torque Maximum TM (BU) obtained were used to assess gluten network strength. The results are presented in Table 5 with the corresponding gluten aggregation curves shown in Figure 1. In white wheat dough, the addition of salt shortened the PMT and increased the TM up to 0.3–0.6% salt in comparison to the salt-free white control (61.3 ± 9.8 s; 56.3 ± 0.6 BU), indicating faster aggregation and a stronger gluten network, whereas 1.2% salt led to a slight increase in PMT (67.7 ± 1.5 s) and a reduction in TM (65.3 ± 2.5 BU) compared to the 0.3% and 0.6% salt samples, suggesting over-tightening of the gluten structure. The GlutoPeak curves for white wheat flour show a typical three-phase pattern—initial, aggregation, and breakdown—as described by Boudrag et al. [23]. For the salt-free white wheat sample, the torque increased slowly and reached a late, broad maximum, consistent with slower gluten aggregation. At 0.3% and 0.6% salt, the torque rise was steeper and the peak occurred earlier, indicating faster aggregation. At 1.2% salt, the curve showed the longest initial phase, followed by a late, high peak. In fibre-fortified dough, adding up to 0.6% salt caused only small decreases in PMT, together with a gradual reduction in TM, compared to the salt-free fibre-fortified sample (121.3 ± 3.8 s; 88.0 ± 6.1 BU). The 1.2% salt sample differed significantly from the salt-free fibre-fortified sample, showing markedly lower PMT (79.3 ± 23.2 s) and TM (59.7 ± 1.5 BU), with a significantly decreased TM compared to the 0.3% and 0.6% salt formulations. At 1.2% salt, PMT showed a comparatively high standard deviation, indicating substantial variability in gluten aggregation, which is in line with the known sensitivity of fibre-rich doughs to small differences in mixing and hydration conditions [41,42]. The gluten aggregation curves for the fibre-fortified white wheat recipes show a distinct pattern, similar to that described by Boudrag et al. [23], with a very short initial phase followed by a long aggregation phase characterised by a plateau-like peak. The initial rise is similar across all salt levels, but the salt-free dough reaches the highest and most prolonged peak. At 0.3 and 0.6% salt, the torque increases more rapidly to a lower peak and then stabilises. At 1.2% salt, the torque reaches the lowest plateau, with a relatively flat curve. The wholemeal dough showed the longest PMT (119.7 ± 5.5 s) when no salt was added, compared to the white wheat formulations, whereas the addition of salt nearly halved this value (≈60 s). Among the three salt-containing formulations (0.3–1.2% salt), no significant differences were detected in either PMT or TM. All gluten aggregation profiles of the wholemeal formulations exhibited a gradual increase in torque, with an initial development phase that was more pronounced than in the fibre-fortified doughs but shorter than in the white wheat control, followed by a broad peak of comparable magnitude across salt levels. The salt-free wholemeal sample displayed an extended aggregation phase with no clearly defined maximum. In contrast, the addition of salt reduced PMT and resulted in a more distinct peak, indicating slightly faster and more pronounced gluten aggregation.

Dough fermentation was studied using a Rheofermentometer, and the corresponding values are reported in Table 5. For each formulation, HM (mm), total CO_2_ produced (mL), volume of CO_2_ lost (mL), volume of retention (mL), and retention coefficient (%) were determined. Height development, CO_2_ production and retention were strongly influenced by both flour type and salt concentration, but the CO_2_ retention coefficient did not differ significantly across the tested flour types and salt levels. For the white wheat control dough, fermentation parameters at 0.3% and 0.6% salt did not significantly differ from those of the salt-free white wheat dough. In contrast, the 1.2% salt formulation exhibited reduced yeast fermentation parameters compared with the other salt levels and the salt-free sample, with lower HM, CO_2_ produced and CO_2_ retained. In the fibre-fortified white wheat formulation, HM was lower than in the white wheat control samples. HM increased significantly between 0.3% and 0.6% salt, then decreased at 1.2% salt, whereas total CO_2_ production and retention volume peaked at 0.3% salt and gradually decreased with further salt addition. In wholemeal dough, HM did not change significantly with salt addition, whereas total CO_2_ produced and retained volume were maximal at 0.3% salt and lowest at 1.2% salt.

### 3.4. Bread Quality Evaluation

The specific volume of the bread loaves was measured, and the results are shown in Table 6. A salt-dependent maximum was observed, which differed slightly among formulations. In white wheat bread, the addition of salt led to a marked increase in specific volume, reaching a maximum of 4.6 mL/g at 0.6% salt before declining to 4.0 mL/g at 1.2% salt, though values remained higher than those of the salt-free control. In fibre-fortified white wheat bread, a 0.3% salt level did not enhance specific volume in comparison to the salt-free bread, whereas a significant increase was observed at 0.6% salt (4.2 mL/g), followed by a reduction to 3.8 mL/g at 1.2% salt, which still exceeded the salt-free sample. In contrast, salt addition showed no significant effect on specific volume in the wholemeal formulations.

Breadcrumb hardness was determined by texture analysis, and the results are presented in Table 6. In white wheat control breads, hardness did not significantly change at 0.3% salt, reached a minimum at 0.6% salt (2.2 N), and increased to a maximum of 5.5 N at 1.2% salt. A similar trend was observed in fibre-fortified white wheat breads, where no significant difference occurred at 0.3% salt, the lowest hardness was recorded at 0.6% salt (4.0 ± 0.9 N), but a non-significant increase was noted at 1.2% salt (5.0 ± 1.1 N). For wholemeal formulations, a significant reduction in hardness was observed at 0.3% and 0.6% salt, with the lowest value of 15.3 N at 0.6% salt. The staling rate of the different formulations, derived from hardness measurements, is presented in Table 6. In white wheat control breads, the addition of salt led to a significant increase in staling rate up to 0.6% salt (4.1 ± 1.7%), followed by a reduction to 2.8 ± 0.3% at 1.2% salt. In contrast, no significant changes in staling rate were observed with salt addition in either the fibre-fortified white wheat or wholemeal formulations.

The number and diameter of cells were determined by C-Cell analysis, and results are found in Table 6. Across formulations, salt level modulated both cell number and diameter, with distinct responses for white wheat, fibre-fortified white wheat and wholemeal breads. At 0% salt, white wheat bread showed relatively large cells (≈2.9 mm) and a moderate number of gas cells (≈3800), whereas fibre-fortified white wheat and wholemeal breads displayed smaller average diameters (≈2.1–2.3 mm) and slightly higher cell counts. Increasing salt to 0.3% reduced cell diameter for all formulations, while the number of cells increased, especially in white wheat and fibre-fortified white wheat breads (to ≈4800–4900 cells). At 0.6% salt, average cell diameter remained similar to 0.3% or increased for the fibre-fortified white wheat formulation. Cell numbers decreased for the white wheat control, grew further for the fibre-fortified white wheat bread, and were maintained in the wholemeal bread. At 1.2% salt, cell diameters tended to decrease (white wheat and wholemeal breads) or remained stable (fibre-fortified white wheat bread), while cell numbers continued to increase or plateau; the fibre-fortified white wheat formulation reached the greatest cell count (≈5600 cells), whereas wholemeal bread showed smaller gains, and white wheat bread showed a non-significant decrease.

Water activity was measured across all formulations, and the results are displayed in Table 6. Water activity of the breads remained high and very similar across formulations and salt levels, with values between 0.96 and 0.98.

Crumb colour difference (ΔE_crumb_) between salt-free and salted formulations was determined for all three flour types, and the results are reported in Table 6. In this context, higher ΔE values indicate a more perceptible visual colour difference from the control, whereas lower ΔE values reflect greater colour similarity. The white wheat breads showed the greatest ΔE_crumb_ overall, with the 1.2% salt formulation reaching the highest value (4.6). In both white wheat control and fibre-fortified white wheat breads, no significant difference in crumb colour was observed between 0.3 and 0.6% salt, whereas ΔE_crumb_ increased at 1.2% salt. For wholemeal breads, which exhibited the smallest ΔE_crumb_ values, a significant difference appears between 0.3% and both 0.6 and 1.2% salt formulations.

## 4. Discussion

Salt markedly affected dough and bread properties, although the extent of the effect depended on flour type (white wheat, fibre-fortified white wheat, or wholemeal). It should be noted that vital wheat gluten was incorporated into the fibre-fortified formulations to compensate for the dilution of gluten associated with flour replacement by fibre ingredients. While supplemental vital gluten may exhibit hydration and aggregation characteristics that differ from those of native wheat gluten, its inclusion level was kept constant across all fibre-fortified formulations. Therefore, the differences observed among salt treatments within the fibre-fortified systems were evaluated under equivalent gluten supplementation conditions. Nevertheless, interactions between salt, fibre components, and supplemental gluten may have contributed to some observed changes in dough and bread properties, and these effects should be considered when interpreting the response of the fibre-fortified formulations. Gluten aggregation in white wheat dough showed a clear dependence on salt concentration, as reflected by GlutoPeak analysis, with gradual improved aggregation at 0.3 and 0.6% salt and a subsequent decrease at 1.2%, indicating that 0.6% represents an apparent optimum salt level for gluten development. Multiple studies have investigated the impact of varying or reducing salt levels on white wheat dough properties. Melnyk et al. [43] evaluated how different salts and ions applied at various concentrations influenced gluten aggregation time and gluten strength using the GlutoPeak test. In line with our results, they reported that torque increased with rising salt concentration, with the most pronounced effects at the highest levels. These authors attributed the changes primarily to modifications of electrostatic interactions in the presence of the added ions: at low concentration, salt ions neutralise charges on amino acids, allowing for the inter-protein aggregation of hydrophobic surface polypeptides and hydrogen bonding, with no impact on the native organisation of gluten proteins. At higher concentrations, they explain that electrostatic forces become negligible and kosmotropic salts such as NaCl strengthen water structure, reducing water available to hydrate gluten, which favours native hydrophobic interactions and limits protein unfolding. Consequently, longer mixing is needed to unfold gluten proteins and promote hydrogen bonding, hydrophobic associations, disulfide exchange, and entanglements required for full gluten development, consistent with an increased PMT, as we observed at 1.2% salt. This concentration-dependent effect of NaCl was also observed in other studies on wheat flour doughs, such as those by Beck et al. [15] and Belz et al. [44], Wang et al. [45], McCann and Day [46], as well as a study by Song et al. [47], who investigated the effects of ion strength on the amyloid fibril formation of rice proteins. In fibre-fortified white wheat dough, PMT values fluctuated with salt addition, with a minimum at 1.2% salt but remaining generally higher than in the white control, which suggests that, in the presence of fibre, the ionic effects of salt on gluten aggregation kinetics are partially masked by water competition and by the structural dilution and disruption of the gluten network already reported in various studies for fibre-enriched systems [23,48,49,50]. At 1.2% salt, the marked reduction in PMT points to the additional disruption of gluten aggregation due to excessive ionic strength. Salt addition did not enhance TM as observed in the white control; instead, TM progressively decreased, suggesting that despite faster aggregation, the effective gluten network strength is reduced. NaCl has been shown to modulate not only gluten–gluten interactions but also the behaviour of charged polysaccharides such as pectin and modified celluloses, by screening their charges and altering their conformation and interactions with gluten, which can weaken or rearrange the limited gluten-gluten contacts, potentially explaining our observations [51]. The reduction in TM at 1.2% salt is consistent with the observations made for the white wheat control sample. Overall, these findings suggest that, in fibre-fortified white wheat dough, salt may reorganise water distribution and polymer–polymer interactions in a crowded system, which could contribute to faster yet less effective gluten aggregation, whereas in white wheat dough NaCl can, up to an optimum concentration, promote more continuous and stronger gluten network formation. In wholemeal dough, the addition of NaCl markedly reduced PMT by half, whereas TM remained essentially unchanged. In such systems, the gluten network is disrupted by particles, and water is more strongly competed for, so changes in ionic environment are more likely to impact aggregation kinetics (PMT) rather than the TM [52,53].

The marked differential response of white wheat, fibre-fortified white wheat and wholemeal doughs to salt reduction likely reflects differences in gluten network architecture and water distribution. In white wheat dough, the observed changes in PMT and TM are consistent with NaCl moderating electrostatic interactions within the gluten network, influencing both aggregation kinetics and network strength. In fibre-enriched and wholemeal systems, insoluble fibre and bran particles disrupt the continuity of the gluten matrix and compete for water, so the technological impact of salt appears attenuated. We propose the concept of “attenuated salt responsiveness”: aggregation kinetics still respond to salt concentration, but network mechanical properties remain largely constrained by the underlying fibre architecture. From a practical standpoint, this suggests that salt-reduction strategies should be formulation-specific rather than universal. In refined white flour systems, careful control of salt level is important, as both insufficient and excessive salt can impair dough handling and bread quality. In fibre-enriched formulations, the disrupted gluten network seems to provide some buffering against salt changes, making these systems potentially more tolerant of larger sodium reductions without proportional deterioration in the technological attributes measured here, though this hypothesis requires further validation.

Yeast fermentation was also affected by salt. In the white wheat control dough, fermentation parameters were altered at the highest salt level. While NaCl up to 0.6% did not significantly impact the fermentation parameters, at 1.2% all indices besides the retention coefficient declined, indicating a clear inhibition of fermentative activity. Similar observations were reported by Pasqualone et al., Lynch et al. and in the review by Belz et al. [17,44,54], who examined the impact of salt reduction on Rheofermentometer parameters in white wheat dough. This behaviour is consistent with reports of osmotic stress imposed by elevated extracellular NaCl, which dehydrates yeast cells (*Saccharomyces cerevisiae*), perturbs membrane function, and slows CO_2_ production [17,55,56]. In addition to impairing yeast fermentation, the disruption of the gluten network at higher ionic force is likely to alter dough extensibility, thereby limiting the maximum dough height achieved during fermentation [17,18,57]. In fibre-fortified white wheat dough, HM values were lower than for the white wheat control dough, likely due to the partial substitution of white wheat flour with dietary fibres, which dilutes the proportion of fermentable carbohydrates available to yeast and simultaneously weakens the gluten network as explained before, thereby reducing dough expansion capacity during proofing [58,59,60]. Increasing salt from 0 to 0.6% in fibre-fortified white wheat dough significantly raised HM while CO_2_ production and retention coefficient remained comparable, suggesting that moderate salt mainly improved the viscoelastic behaviour of the gluten-fibre network, allowing greater dough expansion rather than enhancing CO_2_ production or the overall fraction of CO_2_ retained. At 1.2% salt, both total CO_2_ and HM declined, as explained for the white wheat control dough. HM is consistently lower in wholemeal dough than in the white wheat doughs at each salt level, and is not impacted by salt addition, reflecting the dilution and disruption of the gluten network by bran particles, restricting the air bubbles’ expansion. At 1.2% salt, similarly to the white wheat dough, CO_2_ production decreased.

Salt addition significantly affected bread quality across white wheat and wholemeal formulations. The specific volume of white wheat bread was significantly improved up to 0.6% salt, then decreased at 1.2% salt. These findings are consistent with the patterns obtained from the GlutoPeak and Rheofermentometer measurements, similar to the observations of Pasqualone et al. [54]. In our study, salt levels up to 0.6% were associated with stronger gluten aggregation and higher HM values, whereas at 1.2% all measured parameters deteriorated. In fibre-fortified white wheat bread, 0.3% salt did not have any impact on the specific volume compared to the salt-free sample, but a significant improvement was observed after the addition of 0.6% salt, consistent with improved gluten aggregation and Rheofermentometer observations. Although Rheofermentometer and GlutoPeak parameters for the 0.3% and 0.6% salt fibre-fortified white wheat doughs were comparable, the higher specific volume at 0.6% suggests that differences in gas cell evolution during baking, rather than in fermentation, contributed to the improved loaf specific volume, consistent with studies showing that bubble size distribution can mean that HM does not fully reflect final specific volume [61,62]. In wholemeal bread, salt addition did not significantly affect bread specific volume, which aligns with the measured dough characteristics. GlutoPeak parameters in wholemeal dough showed only minor changes in gluten aggregation across salt levels, and Rheofermentometer HM and gas-related indices were also similar between salt-free and salted doughs. Together, these results indicate that the tested salt range did not sufficiently modify gluten development or fermentation to produce measurable changes in loaf volume, due to the presence of bran fibre inherent to wholemeal flour, which dominates dough and bread structure [63,64].

Regarding texture, salt influenced both crumb hardness and staling rate across the bread formulations. In the control white wheat bread, 0.3% salt did not significantly affect hardness; however, increasing salt to 0.6% was associated with higher specific volume and reduced crumb hardness, indicating that moderate NaCl levels promoted a softer crumb structure. At 1.2% salt, however, specific volume decreased and hardness increased significantly, indicating that higher NaCl may have suppressed yeast activity and tightened the gluten network, thereby contributing to a denser, firmer crumb despite a greater dough strength, as explained earlier. The fibre-fortified white bread exhibited a trend comparable to the white wheat control, showing no significant change in hardness at 0.3% salt, while a significant softening occurred at 0.6%. Low-salt may have improved gluten aggregation and gas production, but the discontinuous gluten network around fibre particles limited crumb softening, so hardness changed little [65]. At the highest salt level, reduced dough extensibility probably increased crumb density, resulting in higher hardness despite the structurally stronger dough [66,67]. In wholemeal bread, hardness stayed high and changed only slightly at 0.6% salt because crumb texture is dominated by the dense bran and fibre-rich matrix, which restricts expansion and elasticity [63]. Because the staling rate was derived from firmness at only two time points (0 and 5 days), it represents an average 5-day firming rate and does not capture the full, potentially non-linear kinetics of staling. The staling rate of fibre-fortified white wheat and wholemeal breads was unaffected by salt concentration, whereas the white wheat control loaves showed a significant salt-dependent change in staling rate, with an increase up to 0.6% salt, followed by a decrease at 1.2%. In the white wheat control, staling rate showed a non-linear response to salt, likely because crumb firming is governed by starch retrogradation and possibly gluten–starch interactions [68]. The pattern at 0.6% salt is consistent with a tighter network and reduced water mobility, which may accelerate retrogradation and staling, whereas 1.2% salt yielded a denser crumb that partly restricted moisture redistribution, lowering the apparent staling rate [69,70]. Conversely, in fibre-fortified white wheat and wholemeal breads, the strong water-binding and matrix-stiffening properties of insoluble fibre and bran dominated crumb firming behaviour, as reported in several studies by Hemdane et al. and Renzetti et al. [71,72], resulting in a staling rate that was insensitive to salt concentration.

Crumb structure analysis revealed a significant effect of salt level, with the extent of these effects differing among the flour types. In white wheat bread, salt significantly increased crumb cell number and slightly reduced cell diameter, in line with the observed improvements in specific volume and hardness [73]. A study by Sun et al. [74] on sodium reduction in bread showed that lower salt weakens the gas phase, leading to fewer retained bubbles and promoting their growth, which aligns with the lower cell counts and slightly bigger cells observed at low salt levels. In fibre-fortified white wheat breads, cell number significantly increased with salt addition, while cell diameter was only slightly impacted, indicating that NaCl primarily promoted the nucleation and stabilisation of numerous bubbles within a fibre–gluten network [74]. In wholemeal breads, salt produced a gradual increase in cell number with little change, or a slight decrease in cell diameter, which, together with the low specific volumes, suggests that bran-induced discontinuities in the gluten network allowed many cells to form but restricted their growth during baking. Overall, the C-Cell data support the interpretation from GlutoPeak and Rheofermentometer measurements that moderate salt levels improve bubble stabilisation and expansion in white wheat and fibre-enriched white wheat doughs, whereas in wholemeal systems salt has a limited ability to overcome the structural constraints imposed by bran.

Breadcrumb water activity remained essentially constant across all tested salt levels (0.96–0.98 for all formulations). This is likely because, at the relatively low NaCl concentrations studied and the high polymer and moisture contents of bread crumb, water binding is dominated by starch, gluten and fibre rather than by salt, so incremental changes in ionic strength have only a minor effect on the amount of “free” water. In addition, competing interactions between hydrophilic and hydrophobic domains within the crumb matrix may further dampen any salt-induced shifts in water activity. From a practical shelf-life perspective, this result suggests that the tested degree of salt reduction would not be expected to adversely affect microbial stability or starch retrogradation behaviour through changes in water activity.

Since NaCl influences the extent of Maillard and caramelisation reactions in baked products, primarily through the modulation of yeast activity, fermentation rates and water availability, colorimetric analysis of the crumb was conducted to quantify these effects [75,76]. The crumb colour difference ΔE_crumb_ between the samples and their salt-free equivalent was measured. ΔE_crumb_ were small across all formulations, indicating that NaCl exerted at most a modest influence on crumb appearance. This limited response is consistent with the fact that, in bread, salt primarily affects browning indirectly by modulating yeast fermentation, substrate availability and water distribution, while crumb colour is more strongly governed by flour type and the presence of pigments and fibre. In particular, the very low ΔE_crumb_ values in fibre-fortified white wheat and wholemeal breads suggest that intrinsic colour from bran and fibre largely overrides any salt-related changes in Maillard and caramelisation reactions within the crumb.

Taken together, the GlutoPeak data showing increased TM and PMT at moderate salt levels in white wheat dough, the more controlled gas production profiles, and the specific volume and hardness results all point to a consistent mechanism, as explained before: at 0.6% salt, electrostatic screening and protein hydration appear to favour the formation of a continuous yet not over-tight gluten network, supporting gas retention and loaf volume while avoiding excessively firm crumb. At 0% salt, weaker gluten aggregation and less controlled fermentation are reflected in lower volume and higher hardness, whereas at 1.2% salt, the stronger ionic environment and altered water distribution may over-stiffen the gluten phase, again increasing firmness and lowering specific volume. In fibre-fortified and wholemeal systems, these salt-dependent effects are attenuated but still observable, suggesting that salt–gluten interactions remain important even in more complex matrices.

**Figure 2:**
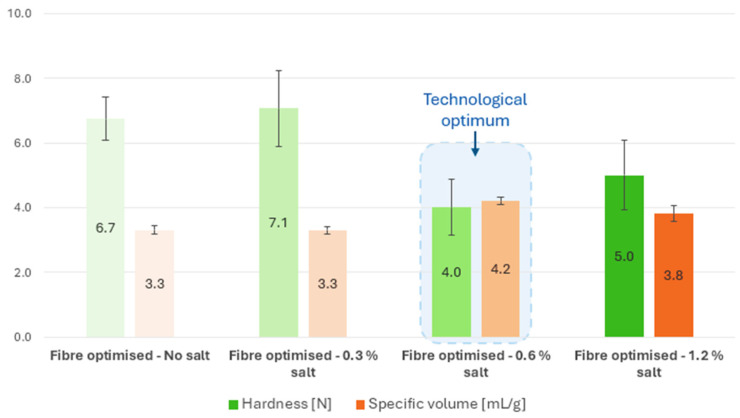
Effect of salt concentration on crumb hardness (N) and specific loaf volume (mL/g) of fibre-fortified white bread. Bars show mean values ± standard deviation. The blue shaded area highlights the technological optimum at 0.6% salt (*w*/*w*). Bar shades of colour distinguish the four salt concentrations indicated on the *x*‑axis.

Across all formulations, 0.6% salt appeared to provide the most favourable overall balance of technological properties. At this concentration, breads generally exhibited higher specific volume and relatively low crumb hardness, acceptable staling rate and a fine, well-aerated crumb structure, without adverse effects on water activity or colour. For the fibre-fortified white wheat bread in particular, these effects were evident as shown in Figure 2, and no detrimental impact on water activity or colour was observed.

For practitioners implementing salt reduction in refined white flour bread, the 0.6% *w*/*w* level may be achievable with minimal process adjustments, but this requires confirmation in industrial bakery trials. Expected outcomes compared to conventional 1.2% formulations, and based on the experimental data, could include the following: (1) nearly identical specific volume and crumb structure; (2) a potentially softer crumb texture (lower hardness at 0.6% salt), which consumer testing should validate; (3) no evidence, within the measured parameters, of deterioration in physical shelf-life indicators; and (4) potential production cost reduction through lower salt usage. Most critically, dough handling characteristics remain consistent, eliminating the need for equipment recalibration or revised mixing protocols.

For fibre-enriched bread systems, the present data suggest an “attenuated salt responsiveness” at the technological level, in the sense that salt reduction produced less pronounced changes in the measured dough and bread quality parameters than in refined white formulations. This interpretation indicates that fibre-enriched matrices may tolerate more substantial sodium reduction without obvious deterioration in the technological attributes assessed here, although this requires confirmation under industrial conditions. Additionally, adaptation of the 0.6% *w*/*w* technological optimal salt level to other bread types (e.g., sourdough, enriched breads) remains an open question warranting investigation. These practical implications should therefore be regarded as preliminary and subject to validation under industrial conditions and with sensory and microbiological assessment.

## 5. Conclusions

The present study demonstrated that the impact of sodium reduction on dough and bread quality is strongly dependent on flour type, with effects being most pronounced in white wheat control breads and less marked in fibre-fortified white wheat and wholemeal systems, where dietary fibres and bran disrupt the gluten network and partially attenuate the functional role of salt. Across all flour types, a salt level of 0.6% (*w*/*w*) emerged as an optimal technological compromise, delivering the best overall balance between dough handling properties and key bread quality attributes while representing a 50% reduction relative to conventional bakery formulations. This is particularly encouraging from a technological standpoint, as it indicates that substantial sodium reduction can be achieved without compromising product performance. Furthermore, a fibre-fortified white wheat bread containing 0.6% salt was successfully developed, integrating two key nutritional strategies: sodium reduction and dietary fibre enrichment. The salt content of this formulation was below the WHO (2021) [10] global sodium benchmark of 0.8% for leavened bread products, underscoring its potential as a health-promoting staple food capable of addressing both inadequate fibre intake and excessive sodium consumption simultaneously. The identification of 0.6% *w*/*w* salt as a technological optimum has direct applicability for commercial bakeries and ingredient suppliers. This salt level is substantially higher than regulatory thresholds for “low-sodium” claims under EU Regulation (EC) No 1924/2006 (which require ≤0.3 g salt per 100 g and ≤0.0125 g salt per 100 g for “low” and “salt-free” claims) yet provides demonstrably superior technological performance compared to ultra-low-salt formulations.

The present study, while comprehensive in scope, is subject to several important limitations that warrant consideration. First, this investigation was conducted under controlled laboratory conditions, utilising standardised recipes and a single yeast strain (*Saccharomyces cerevisiae*), which may not fully reflect the variability encountered in industrial bakery settings, where multiple yeast strains and/or sourdough technology, longer fermentation times, and diverse processing equipment are employed. Second, sensory evaluation and consumer acceptance testing were not conducted; therefore, the acceptability of reduced-salt formulations among end consumers remains unknown, which is critical for market viability. Third, no shelf-life study beyond 5 days was conducted; thus, the long-term stability of bread crumb texture and microbiological safety during typical retail storage (7–14 days) was not evaluated. Finally, economic analysis of reformulation costs and market pricing impact was not included, limiting the assessment of industrial feasibility. These sensory, consumer, and microbiological assessments were outside the scope of the present work and should be addressed in future studies. Despite these limitations, the systematic approach and rigorous methodology provide a robust foundation for identifying optimal salt levels across diverse flour matrices, with results directly applicable to reformulation efforts in research and commercial settings.

## Figures and Tables

**Table 1:** Overview of the 12 bread formulations.

Flour Type	Salt Level (*w*/*w*)
White wheat (control)	0
	0.3
	0.6
	1.2
Fibre-fortified white wheat	0
	0.3
	0.6
	1.2
Wholemeal	0
	0.3
	0.6
	1.2

**Table 2:** Farinograph^®^ water absorption and DDT (Dough Development Time) of white wheat (control), fibre-fortified white wheat and wholemeal doughs at different salt levels. DDT shown as average values ± standard deviation. Different letters in the same column indicate a significant difference across all 12 samples at the level of confidence α = 0.05. Salt levels are expressed in *w*/*w* (g/100 g).

Flour Type	Salt Level (*w*/*w*)	Water Absorption (%)	DDT (min)
White wheat (control)	0	64.4	2.7 ± 0.3 ^a^
	0.3	62.8	2.6 ± 0.1 ^a^
	0.6	61.6	2.4 ± 0.2 ^a^
	1.2	60.3	2.7 ± 0.2 ^a^
Fibre-fortified white wheat	0	88.4	8.0 ± 0.2 ^b^
	0.3	85.6	8.2 ± 0.9 ^bc^
	0.6	84	9.7 ± 0.4 ^cd^
	1.2	83.1	10.8 ± 0.7 ^d^
Wholemeal	0	73.4	9.2 ± 0.3 ^c^
	0.3	72.2	9.4 ± 0.6 ^cd^
	0.6	71.6	9.2 ± 0.6 ^cd^
	1.2	70.3	10.7 ± 0.7 ^d^

**Table 3:** Reduced salt (0.6% *w*/*w*) bread recipes expressed in % of whole recipe (WR) or on flour + fibre basis (FF). BF = baker’s flour (white wheat flour), WMF = wholemeal flour.

Ingredients	White Wheat (Control)		Fibre-Fortified White Wheat		Wholemeal	
Flour Ingredients	%WR	% FF	%WR	%FF	%WR	% FF
Flour (BF or WMF)	58.9	100.0	42.1	82.8	55.6	100
Oat fibre	-	-	3.9	7.6	-	-
Beta-glucan	-	-	2.6	5.2	-	-
Cellulose	-	-	1.5	3.0	-	-
Pectin	-	-	0.7	1.4	-	-
Gluten	-	-	2.2	4.4	-	-
Salt	0.5	0.9	0.5	1.0	0.5	0.9
Sugar	1.2	2.0	1.0	2.0	1.1	2.0
Yeast	1.2	2.0	1.0	2.0	1.1	2.0
Sunflower oil	1.9	3.2	1.6	3.2	1.8	3.2
Water	36.3	61.6	42.7	84	39.8	71.6

**Table 4:** Nutritional composition of white wheat (BF) and wholemeal (WMF) flours as well as white wheat (control), fibre-fortified white wheat and wholemeal breads containing 0.6% (*w*/*w*) salt (reduced salt) used in this study. “—“ signifies not measured.

	BF	WMF	White Wheat Bread (Control)	Fibre-Fortified White Wheat Bread	Wholemeal Bread
Moisture (g/100 g)	14.52 ± 0.30	13.75 ± 0.30	34.8 ± 1.0	40.9 ± 1.2	41.0 ± 1.2
Sodium (mg/kg)	29 ± 17	75 ± 25	2550 ± 460	2450 ± 440	2450 ± 440
Salt (g/100 g)	≈0.0	≈0.0	0.6 ± 0.1	0.6 ± 0.1	0.6 ± 0.1
Proteins (g/100 g) (N × 6.25)	12.59 ± 0.75	15.18 ± 0.82	9.5 ± 0.7	9.3 ± 0.7	10.4 ± 0.7
Fats (g/100 g)	1.59± 0.19	2.19 ± 0.26	2.8 ± 0.2	3.2 ± 0.2	3.4 ± 0.2
Ash (g/100 g)	0.60 ± 0.04	1.34 ± 0.09	1.1 ± 0.1	1.1 ± 0.1	1.5 ± 0.1
Carbohydrates (g/100 g)	70.70 ± 0.83	67.54 ± 0.92	51.7 ± 1.2	45.5 ± 1.4	35.8 ± 2.2
Total Dietary Fibre (g/100 g)	—	—	3.8 ± 0.1	9.3 ± 0.3	8.0 ± 1.7
Energy value (kcal/100 g)	347 ± 2	351 ± 2	270 ± 5	248 ± 5	233 ± 6

**Table 5:** Dough properties shown as average values ± standard deviation. Different letters in the same column indicate a significant difference across all 12 samples at the level of confidence α = 0.05. PMT = Peak Maximum Time; TM = Torque Maximum; HM = Height Maximum.

Flour Type	Salt Level (*w*/*w*)	PMT (s)	TM (BU)	HM (mm)	Total CO_2_ Produced (mL)	Volume of CO_2_ Lost (mL)	Volume of Retention (mL)	Retention Coefficient (%)
White wheat (control)	0	61.3 ± 9.8 ^d^	56.3 ± 0.6 ^e^	63.7 ± 0.3 ^a^	2175 ± 41 ^c^	8.7 ± 0.6 ^ab^	2166 ± 41 ^d^	99.6 ± 0.0 ^ab^
	0.3	45.0 ± 2.6 ^e^	70.7 ± 2.3 ^b^	64.1 ± 0.9 ^a^	2246 ± 77 ^bc^	10.7 ± 1.2 ^a^	2235 ± 78 ^cd^	99.5 ± 0.1 ^a^
	0.6	52.3 ± 2.1 ^d^	71.7 ± 0.6 ^b^	62.8 ± 1.1 ^a^	2158 ± 20 ^c^	6.0 ± 1.7 ^abc^	2152 ± 20 ^d^	99.7 ± 0.1 ^ab^
	1.2	67.7 ± 1.5 ^cd^	65.3 ± 2.5 ^cd^	53.6 ± 1.5 ^b^	1897 ± 45 ^d^	7.3 ± 3.5 ^abc^	1890 ± 44 ^e^	99.6 ± 0.2 ^ab^
Fibre-fortified white wheat	0	121.3 ± 3.8 ^a^	88.0 ± 6.1 ^a^	37.1 ± 2.4 ^d^	2190 ± 82 ^c^	6.3 ± 0.6 ^abc^	2184 ± 84 ^d^	99.7 ± 0.0 ^ab^
	0.3	92.3 ± 6.1 ^ab^	68.7 ± 2.3 ^bc^	50.0 ± 1.8 ^b^	2290 ± 18 ^bc^	5.3 ± 2.1 ^abc^	2285 ± 17 ^bc^	99.8 ± 0.1 ^ab^
	0.6	109.3 ± 17.2 ^ab^	66.0 ± 1.7 ^c^	52.3 ± 1.5 ^b^	2133 ± 51 ^c^	5.7 ± 2.1 ^abc^	2128 ± 51 ^d^	99.8 ± 0.1 ^ab^
	1.2	79.3 ± 23.2 ^bc^	59.7 ± 1.5 ^de^	44.3 ± 3.5 ^c^	1922 ± 48 ^d^	6.7 ± 2.5 ^abc^	1915 ± 46 ^e^	99.7 ± 0.2 ^ab^
Wholemeal	0	119.7 ± 5.5 ^a^	56.0 ± 1.0 ^e^	31.3 ± 1.3 ^e^	2392 ± 74 ^ab^	4.3 ± 1.2 ^bc^	2388 ± 75 ^ab^	99.8 ± 0.1 ^a^
	0.3	60.3 ± 2.5 ^de^	57.3 ± 0.6 ^e^	35.2 ± 2.0 ^de^	2520 ± 33 ^a^	3.3 ± 2.3 ^bc^	2517 ± 31 ^a^	99.8 ± 0.1 ^a^
	0.6	59.3 ± 2.9 ^de^	58.7 ± 0.6 ^de^	36.1 ± 1.0 ^de^	2396 ± 22 ^ab^	3.7 ± 1.5 ^bc^	2392 ± 22 ^ab^	99.8 ± 0.1 ^a^
	1.2	60.0 ± 2.6 ^de^	59.0 ± 1.0 ^de^	34.8 ± 0.1 ^de^	2188 ± 26 ^c^	3.0 ± 1.0 ^c^	2185 ± 26 ^d^	99.9 ± 0.1 ^a^

**Table 6:** Bread properties shown as average values ± standard deviation. Different letters in the same column indicate a significant difference across all 12 samples at the level of confidence α = 0.05. ΔE_crumb_ = crumb colour difference between the sample and its corresponding salt-free control, — signifies “not applicable”.

Sample	Salt Level (*w*/*w*)	Specific Volume (mL/g)	Hardness (N)	Staling Rate (%)	Crumb Cells Number	Cells Diameter (mm)	Water Activity	ΔE_crumb_
White wheat (control)	0	3.8 ± 0.3 ^d^	3.9 ± 1.4 ^g^	2.4 ± 1.0 ^cd^	3757 ± 231 ^f^	2.9 ± 0.3 ^a^	0.98 ± 0.01 ^a^	—
	0.3	4.4 ± 0.3 ^ab^	4.4 ± 0.6 ^fg^	3.5 ± 0.9 ^ab^	4777 ± 349 ^c^	2.6 ± 0.2 ^bc^	0.98 ± 0.01 ^abc^	3.9 ± 0.8 ^ab^
	0.6	4.6 ± 0.2 ^a^	2.2 ± 0.4 ^h^	4.1 ± 1.7 ^a^	4318 ± 242 ^de^	2.7 ± 0.3 ^b^	0.97 ± 0.01 ^bc^	3.0 ± 1.9 ^ab^
	1.2	4.0 ± 0.1 ^cd^	5.5 ± 1.1 ^ef^	2.8 ± 0.3 ^bc^	4273 ± 225 ^de^	2.4 ± 0.2 ^d^	0.97 ± 0.01 ^bc^	4.6 ± 1.1 ^a^
Fibre-fortified white wheat	0	3.3 ± 0.1 ^e^	6.7 ± 0.7 ^d^	1.8 ± 0.3 ^def^	4460 ± 237 ^d^	2.3 ± 0.1 ^d^	0.98 ± 0.01 ^abc^	—
	0.3	3.3 ± 0.1 ^e^	7.1 ± 1.2 ^d^	1.8 ± 0.6 ^cdef^	4934 ± 328 ^c^	2.0 ± 0.1 ^e^	0.98 ± 0.01 ^ab^	1.7 ± 0.1 ^bc^
	0.6	4.2 ± 0.1 ^bc^	4.0 ± 0.9 ^g^	2.1 ± 0.8 ^cde^	5190 ± 258 ^b^	2.4 ± 0.2 ^cd^	0.97 ± 0.01 ^bc^	1.5 ± 0.7 ^bc^
	1.2	3.8 ± 0.2 ^d^	5.0 ± 1.1 ^fg^	2.2 ± 0.3 ^cde^	5648 ± 339 ^a^	2.4 ± 0.3 ^d^	0.98 ± 0.01 ^abc^	2.1 ± 0.1 ^bc^
Wholemeal	0	2.5 ± 0.1 ^f^	20.1 ± 3.0 ^a^	1.1 ± 0.2 ^f^	3786 ± 185 ^f^	2.1 ± 0.1 ^e^	0.97 ± 0.01 ^bc^	—
	0.3	2.7 ± 0.2 ^f^	18.3 ± 2.5 ^b^	1.1 ± 0.3 ^f^	4148 ± 217 ^e^	2.0 ± 0.1 ^e^	0.98 ± 0.01 ^ab^	0.7 ± 0.3 ^c^
	0.6	2.7 ± 0.1 ^f^	15.3 ± 1.9 ^c^	1.2 ± 0.3 ^ef^	4208 ± 263 ^e^	2.0 ± 0.1 ^e^	0.98 ± 0.01 ^abc^	1.8 ± 0.4 ^bc^
	1.2	2.5 ± 0.1 ^f^	19.1 ± 2.1 ^ab^	1.4 ± 0.4 ^ef^	4471 ± 293 ^d^	1.8 ± 0.1 ^f^	0.96 ± 0.00 ^c^	1.7 ± 0.5 ^bc^

## Data Availability

The original contributions presented in this study are included in the article. Further inquiries can be directed to the corresponding author.

## Abbreviations

The following abbreviations are used in this manuscript:

BF	Baker’s flour
DDT	Dough Development Time
HM	Height Maximum
PMT	Peak Maximum Time
TM	Torque Maximum
WMF	Wholemeal Flour

## Appendix A

**Table A1:** Formulations of all bread recipes (except 0.6% salt) expressed in % of whole recipe (WR) or on flour + fibre basis (FF). BF = baker’s flour (white wheat flour), WMF = wholemeal flour.

Ingredients	White Wheat No Salt		White Wheat 0.3% Salt		White Wheat 1.2% Salt		Fibre-Fortified White Wheat No Salt		Fibre-Fortified White Wheat 0.3% Salt		Fibre-Fortified White Wheat 1.2% Salt		Wholemeal No Salt		Wholemeal 0.3% Salt		Wholemeal 1.2% Salt	
Flour Ingredients	%WR	%FF	%WR	%FF	%WR	%FF	%WR	%FF	%WR	%FF	%WR	%FF	%WR	%FF	%WR	%FF	%WR	%FF
Flour (BF or WMF)	58.3	100.0	58.7	100.0	59.0	100.0	41.4	82.8	41.9	82.8	42.1	82.8	55.4	100	55.6	100	55.8	100
Oat fibre	-	-	-	-	-	-	3.8	7.6	3.8	7.6	3.9	7.6	-	-	-	-	-	-
Beta-glucan	-	-	-	-	-	-	2.6	5.2	2.6	5.2	2.6	5.2	-	-	-	-	-	-
Cellulose	-	-	-	-	-	-	1.5	3.0	1.5	3.0	1.5	3.0	-	-	-	-	-	-
Pectin	-	-	-	-	-	-	0.7	1.4	0.7	1.4	0.7	1.4	-	-	-	-	-	-
Gluten	-	-	-	-	-	-	2.2	4.4	2.2	4.4	2.2	4.4	-	-	-	-	-	-
Salt	0	0	0.3	0.4	1.1	1.9	0	0	0.3	0.5	1.0	2.0	0	0	0.3	0.5	1.0	1.8
Sugar	1.2	2.0	1.2	2.0	1.2	2.0	1.0	2.0	1.0	2.0	1.0	2.0	1.1	2.0	1.1	2.0	1.1	2.0
Yeast	1.2	2.0	1.2	2.0	1.2	2.0	1.0	2.0	1.0	2.0	1.0	2.0	1.1	2.0	1.1	2.0	1.1	2.0
Sunflower oil	1.9	3.2	1.9	3.2	1.9	3.2	1.6	3.2	1.6	3.2	1.6	3.2	1.8	3.2	1.8	3.2	1.8	3.2
Water	37.5	64.4	36.9	62.8	35.6	60.3	44.2	88.4	43.3	85.6	42.3	83.1	40.6	73.4	40.1	72.2	39.2	70.3
